# Overcoming Challenges in Providing Radiation Therapy to Patients With Cancer in Nigeria and Experience in the National Hospital Abuja, Nigeria

**DOI:** 10.1200/JGO.19.00177

**Published:** 2020-08-05

**Authors:** Simeon Chinedu Aruah, Obinna Chizoba Asogwa, Fatima I. Ubah, Nandul Nimark Maurice, Rasaaq Oyesegun, Taofeeq A. Ige, C. Norman Coleman, Manjit Dosanjh, David Pistenmaa

**Affiliations:** ^1^ National Hospital Abuja, Abuja, Nigeria; ^2^ International Cancer Expert Corps, Washington, DC; ^3^ Agensi Nuklear Malaysia, Selangor, Malaysia

Globally, cancer is the second leading cause of death among noncommunicable diseases.^1^ It is estimated that the annual global cancer incidence will rise from 17 million cases in 2018 to as many as 27.5 million cases in 2040.^2^ Approximately 65% to 70% of this increase will occur in low- and middle-income countries (LMICs), where cancer is a major cause of morbidity and mortality.^3^ The 66th United Nations General Assembly and the United Nations Sustainable Development Goals have emphasized addressing this burden of noncommunicable diseases.^4^ As expected, the negative economic impact is greater and especially problematic for these countries as the result of premature deaths and loss of years of productivity.^5-7^

**FIG 1:**
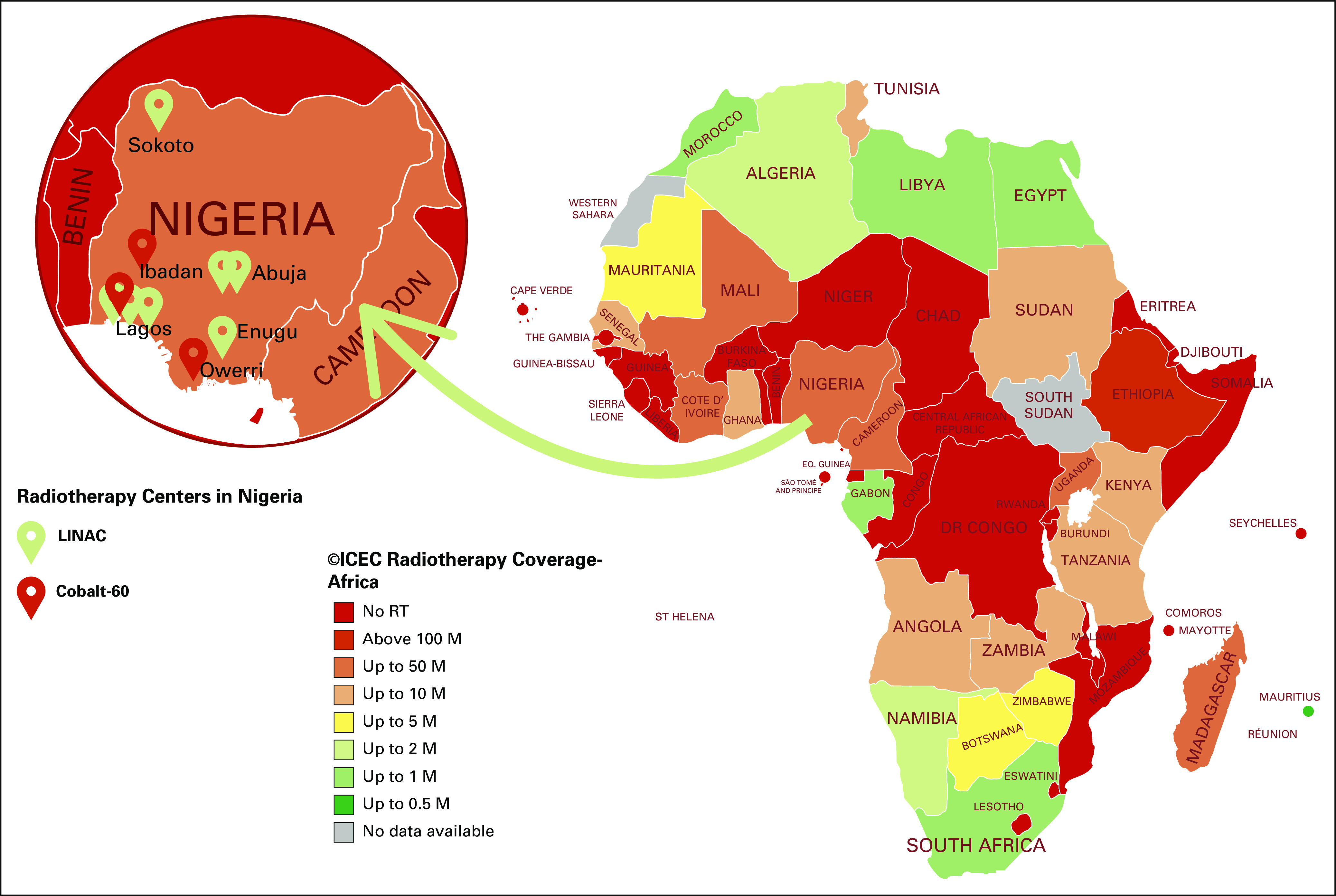
Radiation therapy (RT) coverage in Africa. Map shows the number of people per functioning machine in countries. Only one country has one machine per 0.5 million (M) inhabitants; the rest have much fewer, and 29 countries have no access. The inset highlights Nigeria, the most populous country on the continent, with seven linear accelerators (LINACs) and three cobalt-60 machines. ICEC, International Cancer Expert Corps.

Radiotherapy has a role in the cure or palliation of more than 50% of patients with cancer. Abdel-Wahab et al,^8^ Barton et al,^9^ and Atun et al^10^ argue that radiotherapy is a critical and cost-effective component of a comprehensive cancer control plan. In LMICs, there is a severe shortfall in radiation treatment capacity, especially in Sub-Sahara Africa.^11^ It is estimated that 1.05 million new cases of cancer and 693,000 deaths occurred in Africa in 2018^3^ and that there will be more than 1.5 million new cases of cancer in Africa in 2025.^12^ According to the Directory of Radiotherapy Centres database,^13^ the 160 radiotherapy centers in Africa are equipped with a total of 189 linear accelerators (LINACS) and 88 cobalt-60 machines, with more than 60% in South Africa, Egypt, and Morocco.^11^ This shortfall in radiation treatment capacity in Africa as a whole, and specifically in Nigeria, is illustrated in Figure 1. Nigeria has an estimated population of 200 million people^14^ and experiences approximately 115,000 new cases of cancer and around 70,000 cancer deaths per year.^15^ Yet it has only seven LINACs: three at Lagos University Teaching Hospital (LUTH), Lagos; two at National Hospital Abuja (NHA), Abuja; and one each at Usmanu Danfodiyo University Teach-ing Hospital, Sokoto, and University of Nigeria Teach-ing Hospital, Enugu. Nigeria also has three cobalt-60 machines: one each at University College Hospital, Ibadan, Eko Hospital, Lagos, and Imo International Health Systems, Owerri. On the basis of the International Atomic Energy Agency criterion of one LINAC megavoltage machine for every 400 patients with cancer (22), Nigeria has a shortfall of approximately 280 LINACS. There are comparative advantages of LINACS over cobalt-60 machines, as documented by Efstathion et al^16^ and Healy et al.^17^ However, a large number of cobalt-60 units are still in use in Africa, primarily for economic reasons. Current concerns in Africa regarding cobalt-60 units, half of which are more than 20 years old,^18^ are the requirements for security of the radioactive sources and the ever-increasing costs for their disposal.^19^

Efforts to improve access to radiotherapy in Nigeria began in 1968 when LUTH acquired a superficial kV radiotherapy machine. In 1973, LUTH became the first center in West Africa to acquire a cobalt-60 machine. In the 1980s, the privately owned radiotherapy center in Eko, Hospital, Lagos, also acquired a cobalt-60 machine. In the early 2000s, Nigeria acquired five LINACs. The first was installed in 1999 at NHA, a multidisciplinary hospital with more than 400 beds and various specialist care units, including radiation therapy and clinical oncology. This was followed by the installation of LINACs in radiation therapy centers in Enugu, Sokoto, Lagos, and Benin Teaching Hospitals (Fig 1).

Common problems experienced by LINAC users in Nigeria lead to long waiting times for patients. These include difficulty diagnosing machine problems, administrative delays in acquiring spare parts, and lack of technical support to maintain and repair the LINACS. Fortunately, in Abuja, we had an ingenious engineer who kept our initial LINAC running much of the time. Often it was the only functional LINAC in Nigeria. However, the facilities in Sokoto and Enugu, especially after the latter became a public–private partnership, also were able to function intermittently despite the challenges. NHA also maintained a high level of power production that overcame the frequent power failures experienced by other radiation oncology facilities in Nigeria.

In 2017, the original LINAC in Abuja was decommissioned because the manufacturer no longer provided spare parts. NHA had purchased an Elekta Synergy LINAC capable of delivering three-dimensional conformal radiation therapy/intensity-modulated radiation therapy/volumetric-modulated arc therapy in conjunction with a computerized treatment planning system, a Record and Verify system, a 64-slice computed tomography simulator, as well as comprehensive physics and dosimetry equipment in 2014. However, the LINAC was not installed and commissioned until the shielded bunker was completed in 2017. In 2019, a new Elekta Agility LINAC with a 160-leaf multileaf collimator was donated to NHA. It was immediately installed and commissioned; it started being used to treat patients by the end of the year. NHA also acquired a standby generator to ensure a steady power supply if the electrical grid failed. The enlarged radiation therapy staff includes biomedical engineering support arising from a formalized service contract with the manufacturer’s representative in the country. These improvements have reduced machine downtime and patient waiting times, allowing NHA to increase its radiation treatment capacity to 80 to 100 patients daily.

At NHA, we learned that there are many barriers to establishing a functional LINAC-based radiation therapy center in a challenging environment, such as ours in Abuja. On the basis of our interactions with other radiation therapy centers in Nigeria and with participants at international workshops, we learned that our experience at NHA mirrors the experiences of radiation therapy centers elsewhere in Nigeria and Africa and in other low-resource countries. We are pleased to make a number of observations regarding the establishment of LINAC-based radiotherapy centers under challenging circumstances and offer recommendations pertaining to support from local, regional, and federal governments.

One of the greatest challenges in the use of radiotherapy in Nigeria is the broad lack of human resources including radiation oncologists, medical physicists, dosimetrists, oncology nurses, radiation therapy technologists, and LINAC maintenance engineers. A major aspect of technology transfer is establishing a means for the ongoing transfer of expertise to staff in low-resource countries. For example, NHA has two LINACS that are capable of intensity-modulated radiation therapy and volumetric-modulated arc therapy. However, because of inadequate training, we are unable to perform those advanced radiation treatment techniques.

We are pleased that our federal government recently has increased our treatment capacity and capabilities at NHA. In addition, at the Nigerian Sovereign Investment Authority/LUTH center, a public–private partnership arrangement has provided a cobalt-60 machine at Imo International Health Systems, Ikeduru, as well as high-dose rate machines for several centers. Still, there is a tremendous need for additional financial support from local and regional governments, as well as from our federal government, to overcome the great shortfall of treatment capacity with radiation therapy. This shortfall stems not only from the lack of LINACS or cobalt-60 units but also the supporting technologies such as diagnostic imaging, clinical and anatomic pathology, and nuclear medicine imaging. It is now recognized that safe and effective radiotherapy services need both of the following: (1) substantial capital investment in radiotherapy equipment and specially designed facilities, and (2) continuous investment in maintenance and upgrading of the equipment in line with technical progress. One way to circumvent the limitations of governmental funding for radiation therapy centers is to encourage formulation of policies to stimulate formation of public–private partnerships in Nigeria and other LMICs. In addition, a national plan for reimbursement of radiation treatments for patients with cancer would provide financial sustainability for cancer programs. This was recently introduced by the Nigerian National Health Insurance Scheme, although the impact of this initiative is yet to be felt in the various treatment outlets.

There is a great need to develop a Nigerian national cancer registry, as called for in the National Cancer Control Plan.^20^ In the absence of a national cancer registry, most of the available statistics on cancer incidence, prevalence, and mortality are based on estimates. Available cancer hospital–based data are not reliable enough for national planning. A national cancer registry is urgently needed to collect and continuously update data for epidemiologic studies, as well as to study the delivery and outcome of cancer care.

Nigeria lacks efficient referral systems for patients with cancer, especially for those patients who would benefit from radiotherapy if they were seen earlier in the course of their disease. In large part, this is because of the lack of awareness of cancer in the general population. This situation is supported by studies done by Grover et al^21^ and Aruah et al,^22^ which showed that more than 60% of patients with cancer in LMICs presented with locally advanced disease.

LINACS require a steady power supply to perform reliably. Nigeria has yet to have a stable power supply throughout the country, thereby often adversely affecting the performance of treatment machines and lengthening patients’ waiting times. To ensure reliable performance of high-tech equipment in radiotherapy centers throughout Nigeria, it is essential to provide the necessary infrastructure. This would include stable power and water supply, excellent physical buildings to house the radiotherapy machines, improved means of transportation for patients to access treatment, and provision of security for facilities that still have cobalt-60 machines.

We recommend the provision of funds to establish a national cancer institute that would become the leading focus of cancer research and treatment of future generations of Nigerians. This would stimulate the integration of available research and information related to the planning and delivery of quality service, including radiation therapy, for patients with cancer in Nigeria. The recommended increase in financial support outlined above is consistent with the budgetary allocations to health and education on national and regional levels needed to meet WHO recommendations regarding patient care and research. The establishment of radiotherapy centers under the direction of a national cancer institute could support local and regional health care programs by providing focal points to address other noncommunicable diseases as well as infectious diseases.

We have also learned that there are many considerations on the local and regional levels that influence the further development of radiotherapy centers in challenging environments, such as that in which several of us work. In addition to having sufficient numbers of well-trained radiotherapy staff and up-to-date technology (both hardware and software) to provide high-quality radiotherapy care to patients with cancer, it is important to create a positive work environment. This includes an organizational culture (supported by effective leadership and management) that promotes smooth collaboration among multidisciplinary teams in all aspects of patient care. There should be a strong culture of continuous quality improvement using robust audit tools as needed to support evidence-based practice and to drive quality forward. Achieving recognized levels of multidisciplinary care and developing sufficient treatment capacity will allow facilities to manage a greater patient volume. This will thereby create economies of scale to sustain the radiation therapy programs. Increased investment in translational research, with transfer of this knowledge into practice, is needed to enable better treatments and to achieve global standards of patient care and research.

In summary, this commentary highlights the shortfall in radiotherapy capacity in Africa, particularly in Nigeria, which is the most populous country on that continent. It shows that having recognized the challenge presented by the increasing incidence of cancer, Nigeria is striving to increase access to radiotherapy, taking into consideration the comparative advantages of LINACs over cobalt-60 machines. The continuum of cancer care needed is well established in high-income countries’ approaches to global mentorship. This helps to establish high-quality programs and creates partnerships with world-renowned experts that enhance our spirit and bring us into professional partnerships and collaboration.^23^ This commentary also enumerates the challenges faced by the authors and their successful efforts to implement treatment with LINACs at NHA. We present recommendations that may help institutions in other LMICs that are contemplating the purchase of an LINAC. We believe that dedicated governmental support and motivated staff can cut the costs associated with the operation and maintenance of LINACs, thereby giving patients with cancer the best option for radiotherapy treatment. We must match the increasing cancer prevalence with a growing level of effective use of new technology, including modern LINACs. This is an opportunity for innovative systems solutions to include developments in technology and transfer of expertise through education and sustainable mentoring to enable advanced treatment capacity. This, we believe, is the best way to win the race against cancer.
